# Supplementary material for: Does Exercise Training Improve Cardiac-Parasympathetic Nervous System Activity in Sedentary People? A Systematic Review with Meta-Analysis
Source: Int J Environ Res Public Health. 2022 Oct 26;19(21):13899. doi: 10.3390/ijerph192113899 (PMC9656115; doi:10.3390/ijerph192113899)
Supplement: Supplementary file 1 [file ijerph-19-13899-s001.zip › ijerph-1870483-supplementary.pdf]

ELECTRONIC SUPPLEMENTARY MATERIAL

Section 1: Electronic database search terms.

(2) Interventions:

#1 Exercise OR training OR HIIT OR MCT: Ti/ab

(3) #1 AND #2

(4) Outcomes:

#4. “heart rate variability” OR HRV OR “heart rate recovery” OR HRR OR parasympathetic OR vagal OR “autonomic function” OR “autonomic modulation” OR “autonomic tone”: Ti/ab

(5) #3 AND #4

Section 2: Tables.

Table S1. Criteria used to carry out methodological quality assessment.

**Item**	**Question**	**Additional Information**	**Scoring**
		**Study Quality**	
1	Eligibility criteria specified	Eligibility criteria should be specified and fulfilled, and specific diagnostic test values should be provided for all participants.	Yes (1) /No (0)
2	Allocation concealment	It should be stated if group allocation was concealed; meaning if a patient was eligible for inclusion in the trial was unaware (when this decision was made) of which group the patient would be allocated to. Yes, if group allocation was concealed from patients eligible for inclusion in the trial (e.g. consent should be given before randomization).	Yes (1) /No (0)
3	Randomization specified	A description of the method used to allocate patients into treatment groups should be provided. Yes, if methods are described and they are truly random e.g., coin-tossing, sequence of randomly generated numbers.	Yes (1) /No (0)
4	Groups similar at baseline	Baseline data of all participants who were randomized should be presented. There should be no significant difference in the measure of the severity of the treated condition between treatment groups. 1 Point – if baseline data are separated by group allocation, presented and no differences are apparent.	Yes (1) /No (0)
5	Blinding of assessor for at least one key outcome	It is not always possible to blind patients and/or therapists; however, blinding of assessors is reasonable. If assessors of primary outcome measures are blinded to the intervention allocation of the patients, this should be stated clearly.	Yes (1) /No (0)

Table S1. Continued.

Item	Question	Additional Information	Scoring
		**Study Reporting**	
6	Assessment of outcome measures	The percentage of patients completing the study in both groups should be reported. 1 Point if adherence >85%, 1 point if adverse events are reported and 1 point if exercise attendance is reported.	Yes (3) /No (0)
7	Intention-to-treat analysis	When a patient withdraws, this analysis is conducted by using either the last value obtained for each of the outcome measures as a post-intervention value, or by using the baseline value as a post value. This analysis should be added to the data of those that did complete the study and an analysis conducted. 1 point for no withdrawal.	Yes (2) /No (0)
8	Between-group statistical comparisons reported	Comparison of exercise vs. comparator (control) group for the primary and at least one secondary outcome should be performed. 1 Point if between-group statistical comparisons are reported for the primary outcome measure of interest, 1-point if f between-group statistical comparisons are reported for at least one secondary outcome measure.	Yes (1) /No (0)
9	Point measures and measures of variability for all reported outcome measures	Point estimates should be provided for all outcomes, otherwise this could be deemed selective outcome reporting.	Yes (1) /No (0)
10	Activity monitoring in control groups	Between-group differences may be diluted if control patient's crossover to intervention. As many as one third of patients do this, so some measure e.g., exercise diary or activity monitoring should be supplied so this effect can be measured and quantified. 1 point if control patients are asked to report their levels of physical activity and data are presented.	Yes (1) /No (0)
11	Relative exercise intensity remained constant	Exercise intensity is considered by many to be the best stimulus for adaptation. Once patients begin an exercise programme at a set intensity they will begin to adapt. Throughout the study duration the relative intensity will fall in those that do adapt. Therefore, periodic assessment of exercise capacity should be conducted, and the intensity titrated up (or in those that lose fitness, titrated down) so that exercise intensity remains constant. 1 point where attempt is made to keep relative intensity constant/ absolute intensity progressive.	Yes (1) /No (0)
12	Exercise volume and energy expenditure reported	Exercise parameters: session and programme duration, session frequency, exercise training intensity and modality should be clearly reported.	Yes (1) /No (0)

Table S2. Intervention and resting heart rate variability assessment characteristics.

Study (author)	Group; exercise type (aerobic method)	Intervention characteristics		HRV assessment characteristics	
		Setting; length; sessions a week	Session details	HRV index; power spectral density method; device	Setting; assessment position; breathing rate
Audette et al. [65] *	IG;<br>AT (MIT)<br><br>CG; NA	Supervised; 12 weeks; 3 sessions	15 min WU (with flexibility) + 40 min walking at 50-70% HR peak (estimated) / Progression: 10 - 30 min 8 weeks / maintained 4 weeks + 5 min CD	HF (ms ^{2}); NR; ECG	Laboratory-based measures; NR; NR
Boutcher et al. [66]	IG;<br>AT (HIIT)<br><br>CG; NA	Supervised; 12 weeks; 3 sessions	5 min WU + 20 min at 80 - 85% HR peak (continuous 8 s sprint cadence 100 and 130 rpm, 12 s recovery cadence 30 rpm) + 5 min CD	HF (ms ^{2}); FFT; HR monitor	Laboratory-based measures; supine; spontaneous
Lopes et al. [77]	IG;<br>RT (NA)<br><br>CG; NA	Supervised; 12 weeks; 3 sessions	10 min pedaling cycle ergometer / 5 min stretching WU + 8 exercises lower and upper body (Week 1, 1 set for exercise a 12-20 rep / Week 2, 3, 4 and 5, 2 sets for exercise 12-20 rep / Week 6, 3 sets for exercise and 12-20 rep. Week 7, 8, 9, 10 and 11, 2 sets for exercise and 12-20 rep / Week 12, 1 set for exercise and 12-20 rep / 50% at 1RM loading adjustments week 4, 7 and 10)	RMSSD; NA; ECG	Laboratory-based measures; supine; 12 breaths per min
Melanson and Freedson [78]	IG;<br>AT (MIT)<br><br>CG; NA	Supervised; 16 weeks; 3 sessions	30 min (cycle) at 70-80% of HRres / cadence 80 rpm	RMSSD and HF (ms ^{2}); FFT; ECG	Laboratory-based measures; supine; 10 breaths per min
Mourot et al. [79]	IG;<br>AT (HIIT)<br><br>CG; NA	NR; 6 weeks; 3 sessions	9 consecutive periods of 5 min including / 4 min “base” work W-VT (ventilatory threshold power output) + 1-min “peak” work W-M (maximal power output)	RMSSD and HF (ms ^{2}); CGSA; ECG	Laboratory-based measures; supine and 60° upright; NR

Table S2. Continued.

Study (author)	Group; exercise type (aerobic method)	Intervention characteristics		HRV assessment characteristics	
		Setting; length; sessions a week	Session details	Study (author)	Group; exercise type (aerobic method)
Oliveira-Junior et al. [80]	IG; CT (HIIT)	Supervised; 12 weeks; 2 sessions	5 min at 60–70% at HRmax in the cycle ergometer WU + 9 exercises in 3 mini-circuit stations / 55 s (60–70% HRmax), 5 s maximal sprint (no load), 30 s maximal sprint / 3 min cycle ergometer (no load; 50–70 rpm) + 5 min no load cycle ergometer / 9 min active stretching (1 rep x 45 s per position, including muscle trunk, upper and lower limbs) CD	RMSSD and HF (ms^2); FFT; HR monitor	Laboratory-based measures; supine and sitting; spontaneous
	CG; NA				
Boutcher and Stein [67]	IG; AT (MIT)	Supervised; 8 weeks; 3 sessions	0.25-mile walking (with flexibility) WU + 20 min walking-jogging-cycling at 60% HRres / Progression: 20 min for the first 3 sessions, 25 min for the next 3 sessions, and 30 rain from the 9th through to the 25th sessions + 0.25-mile walking (with flexibility) CD	HF (ms^2); NR; ECG	Laboratory-based measures; seated; 7.5 breaths per min
	CG; NA				
de Rezende Barbosa et al. [68]	IG; AT (MIT)	Supervised; 18 weeks; 3 sessions	Circuit: 3 x 11 exercise stations (functional training x 30-70 s / 30 s recovery + walk 30 - 18 min	RMSSD, HF (ms^2) and SD_1; FFT; HR monitor	Laboratory-based measures; supine; spontaneous
	CG; NA				
Hautala et al. [69]	IG; AT (MIT)	Supervised; 2 weeks; 5 sessions	5 min WU (cycling 50-W and 75-W resistance for women and men, respectively) + 30 min cycling at 70 – 80% HRmax + 5 min (cycling 50-W and 75-W resistance for women and men, respectively) CD	HF (ms^2); AR; HR monitor	Nocturnal; laying; NA
	CG; NA				
Heydari et al. [70]	IG; AT (HIIT)	Supervised; 12 weeks; 3 sessions	5 min WU + 20 min of 8-s sprint and a 12-s recovery (workload 80 – 90% HRmax cadence 120 and 130 rpm / recovery cadence 40 rpm) + 5 min CD	RMSSD and HF (ms^2); NR; HR monitor	Laboratory-based measures; supine; spontaneous
	CG; NA				
Jelinek et al. [71] *	IG; AT (MIT) HV	Home-based; 8 weeks; 6 sessions	60 min at 70 – 80% HRmax walking and jogging.	SD_1; NA; HR monitor	Nocturnal; laying; NA
	IG; AT (MIT) LV	Home-based; 8 weeks; 6 sessions	30 min at 70 – 80% HRmax walking and jogging.		
	CG; NA				

Table S2. Continued.

Study (author)	Group; exercise type (aerobic method)	Intervention characteristics		HRV assessment characteristics	
		Setting; length; sessions a week	Session details	HRV index; power spectral density method; device	Setting; assessment position; breathing rate
Kanegusuku et al. [72] *	IG; RT (NA)	Supervised; 16 weeks; 2 sessions	7 exercise / Weeks 1–4: sets 2 and 10 rep / Weeks 5–8: sets 2 + 1 and 10 + 8 rep / Weeks 9–12: sets 3 and 8 rep / Weeks 13–14: sets 2 + 2 and 8 + 6 rep / Weeks 15–16: sets 2 + 2 and 6 + 4 rep / 3 min rest sets and exercises	HF (nu); AR; ECG	Laboratory-based measures; seated; NR
	CG; NA				
Karavirta et al. [73]	IG; AT (MIT)	Supervised; 21 weeks; 2 sessions	15 min WU + cycling training - first cycle 7 weeks (30 min below aerobic threshold) / second cycle 7 weeks (one session 45 min, 10 min interval between aerobic-anaerobic thresholds and a 5 min interval above anaerobic threshold - other session 60 min below aerobic threshold) / last cycle 7 weeks (one session 60 min, two 10 min intervals between the aerobic-anaerobic thresholds, two 5 min intervals above anaerobic threshold, and 30 min below aerobic threshold - other session 90 min steady pace below aerobic threshold) + 15 min CD	HF (ms ^{2}); NR; HR monitor	Laboratory-based measures; supine; spontaneous
	IG; RT (NA)	Supervised; 21 weeks; 2 sessions	7 – 10 exercises lower and upper body / first cycle 7 weeks (40–60% of 1RM; 12–20 rep; 3 sets) / second cycle 7 weeks (60–80% of 1RM; 5-12 rep; 2-4 sets)/ last cycle 7 weeks (70-85 of 1RM; 5-8 rep; 2-4 sets)		
	IG; CT (MIT)	Supervised; 21 weeks; 4 sessions	described in the preceding cells		
	CG; NA				

Table S2. Continued.

Study (author)	Group; exercise type (aerobic method)	Intervention characteristics		HRV assessment characteristics	
		Setting; length; sessions a week	Session details	HRV index; power spectral density method; device	Setting; assessment position; breathing rate
Karavirta et al. [74]	IG; AT (MIT)	Supervised; 21 weeks; 2 sessions	15 min WU + cycling training - first cycle 7 weeks (30 min below aerobic threshold) / second cycle 7 weeks (one session 45 min, 10 min interval between aerobic-anaerobic thresholds and a 5 min interval above anaerobic threshold - other session 60 min below aerobic threshold) / last cycle 7 weeks (one session 60 min, two 10 min intervals between the aerobic-anaerobic thresholds, two 5 min intervals above anaerobic threshold, and 30 min below aerobic threshold - other session 90 min steady pace below aerobic threshold) + 15 min CD	HF (ms^2); AR; HR monitor	Laboratory-based measures; supine; spontaneous
	IG; RT (NA)	Supervised; 21 weeks; 2 sessions	7 – 10 exercises lower and upper body / first cycle 7 weeks (40–60% of 1RM; 15–30 rep; 3 sets); second cycle 7 weeks (60–80% of 1RM; 6-12 rep per set); last cycle 7 weeks (70-85 of 1RM; 5-8 rep per set)		
	IG; CT (MIT)	Supervised; 21 weeks; 4 sessions	described in the preceding cells		
	CG; NA				
Kim et al. [75] *	IG; AT (MIT) 1	Supervised; 8 weeks; 3 sessions	5 min WU + 30-50 min on a treadmill at 75% HRres + 5 min CD	RMSSD, HF (ms^2) and SD_1; FFT; HR monitor	Laboratory-based measures; seated; 15 breaths per min
	IG; AT (MIT) 2	Supervised; 8 weeks; 2 sessions	5 min WU + 30-50 min on a treadmill at 60% HRres + 5 min CD		
	CG; NA				
Lee et al. [76] *	IG; AT (MIT)	Supervised; 2 weeks; 4 sessions	40 min cycling / 5 min WU (cycling no added resistance) + 30 min at 80–85% HRres + 5 min CD (cycling no added resistance)	HF (nu); FFT; ECG	Laboratory-based measures; supine and head-up tilt; 12 breaths per min (controlled and spontaneous)
	CG; NA				

Table S2. Continued.

Study (author)	Group; exercise type (aerobic method)	Intervention characteristics		HRV assessment characteristics	
		Setting; length; sessions a week	Session details	HRV index; power spectral density method; device	Setting; assessment position; breathing rate
Rezende Barbosa et al. [81]	IG; RT (NA)<br>CG; NA	Supervised; 12 weeks; 3 sessions	30-40% 1RM (first 3 weeks), 4th week recuperation (untrained), 5th to 8th week ranged from 40-90% of 1RM, 9th week recuperation (untrained) and 10th, 11th and 12th week ranged from 80-100% 1RM.	RMSSD, HF (ms^2) and SD_1; NR; HR monitor	Laboratory-based measures; supine; spontaneous
Romagnoli et al. [82] *	IG; AT (MIT)<br>CG; NA	NR; 8 weeks; 3 sessions	60 min 65 to 80% HRmax theoretical	HRV was not assessed (NA)	HRV was not assessed (NA)
Rossi et al. [83]	IG; CT (HIIT)<br>CG; NA	Supervised; 16 weeks; 3 sessions	5 min WU + 50 min resistance training (9 exercises; 12 - 15 rep 1RM) / 30 min aerobic (anaerobic threshold, 70% HRmax) + 5 min CD	RMSSD and HF (ms^2); FFT; HR monitor	Laboratory-based measures; supine; spontaneous
Shen and Wen [84]	IG; AT (MIT)<br>CG; NA	Supervised; 10 weeks; 3 sessions	90 min per session / 10–15 min stretching WA + step aerobic exercise: target HR first 5–10 min and held 35–40 min (75–85% HRres) + 10-15min balance and CD + 10–15 stretching and relaxation	RMSSD and HF (nu); WD; ECG	Laboratory-based measures; NR; NR
Shiotani et al. [85]	IG; AT (MIT)<br>CG; NA	Supervised; 8 weeks; 3 sessions	35 min ergometer cycling at 60% HRres	HF (ms^2); FFT; ECG	Nocturnal; laying; NA
Sloan et al. [86] *	IG; AT (MIT)<br>CG; NA	Supervised; 12 weeks; 4 sessions	10-15 minutes WU + 30-40 minutes at 55-65% weeks 1-2 / 65-75% weeks 3-4 / 80% HRmax remaining weeks + 10-15 min CD	RMSSD and HF (ms^2); FFT; ECG	Laboratory-based measures; NR; NR

Table S2. Continued.

Study (author)	Group; exercise type (aerobic method)	Intervention characteristics		HRV assessment characteristics	
		Setting; length; sessions a week	Session details	HRV index; power spectral density method; device	Setting; assessment position; breathing rate
Soltani et al. [87]	IG;<br>AT (MIT) HV	Supervised; 12 weeks; 3 sessions	45 min at 50% VO_2max first 2 weeks / 50 min at 55% VO_2max followed 6 weeks (3–7) / 60 min at 60% VO_2max final 5 weeks (8–12)	RMSSD and HF (ms^2); FFT; HR monitor	Laboratory-based measures; supine; spontaneous
	IG;<br>AT (MIT) LV	Supervised; 12 weeks; 3 sessions	25 min at 70% VO_2max first 2 weeks / 35 min at 80% VO_2max followed 6 weeks (3–7) / 40 min at 85% VO_2max final 5 weeks (8–12)		
	CG; NA				
Songsorn et al. [88]	IG;<br>AT (HIIT)	Supervised; 6 weeks; 3 sessions	60 min 65 to 80% HRmax theoretical	RMSSD and HF (ms^2); NR; HR monitor	Laboratory-based measures; supine; 12 breaths per min
	CG; NA				
Tulppo et al. [89]	IG;<br>AT (MIT) HV	NR; 8 weeks; 6 sessions	60 min at 70-80% HRmax walking and jogging	HF (ms^2); AR; HR monitor	Nocturnal; laying; NA
	IG;<br>AT (MIT) LV	NR; 8 weeks; 6 sessions	30 min at 70-80% HRmax walking and jogging		
	CG; NA				
Verheyden et al. [90]	IG;<br>CT (MIT)	Supervised; 48 weeks; 2.5 sessions	75 min / first two sessions familiarizing, two exercise first bicycle ergometry and then treadmill walking/jogging or rowing ergometry at 65 increased gradually to 80% HRres (first 6 months, increased 12 min in the first sessions to 15 min, which was maintained rest year) / 7 exercises (upper and lower limbs) 2 sets of 30 rep 30-RM increased to 20-RM	HF (ms^2); FFT; ECG	Laboratory-based measures; supine and standing; spontaneous
	CG; NA				

AR, auto-regressive; AT, aerobic training; CD, cool-down; CGSA, coarse graining spectral analysis; CT, combined aerobic and resistance training; CG, non-exercise control group; ECG, electrocardiogram; FFT, fast-Fourier transform; HF, high frequency; HIIT, high-intensity interval training; HV, high volume; HR, heart rate; HRmax, maximal heart rate; HRres, heart rate reserve; HRV, heart rate variability; IG, intervention group; LV, low volume; MIT, moderate intensity training; ms^2, milliseconds squared; NA, no applicable; NR, no reported; NU, normalised units; PHR, peak heart rate; 1RM, One-repetition maximum; RMSSD, the root-mean-square difference of successive normal R-R intervals; RPM, revolutions per minute; Rep, repetitions; RT, resistance training; Sc, stretching; SD_1, the standard deviation of instantaneous beat-to-beat R-R interval variability; VO_2max, maximal oxygen uptake; WD, wavelet decomposition; WU, warm-up;

[$]Other details are provided in the aerobic training group

*Excluded from the quantitative synthesis

Table S3. Heart rate recovery assessment characteristics and outcomes reported.

Study (author)	Characteristics		Reported outcomes	
	Test characteristics	Recovery characteristics	HRR index	SMD (95% CI)
Hautala et al. [69]	Cycling; maximal incremental test until volitional exhaustion	Passive; 1 min; seated	HRR 1 min	0.31 (–0.12, 0.74)
Mourot et al. [79]	Cycling; submaximal constant test at 50% VO_2 max; 6 min	Passive; 2 min; NR (position)	T30	–1.42 (–2.47, –0.37)
			T120	–2.61 (–4.03, –1.18)
Romagnoli et al. [82] *	Cycling; submaximal constant test at 65% HR max; 3 min	Passive; 3 min; seated	HRR 1 min	2.02 (1.04, 3.01)
			HRR 2 min	1.43 (0.58, 2.28)
			HRR 3 min	1.62 (0.73, 2.52)
			T30	–0.82 (–1.57, –0.08)
			T30 min	–1.22 (–2.03, –0.41)
			MEF A0	–0.97 (–1.74, –0.20)
			MEF Amax	2.52 (1.40, 3.65)
	Cycling; submaximal constant test at 80% HR max; 3 min	Passive; 3 min; seated	HRR 1 min	1.02 (0.25, 1.80)
			HRR 2 min	1.58 (0.69, 2.46)
			HRR 3 min	2.05 (1.06, 3.05)
			T30	–0.27 (–0.96, 0.43)
			T30 min	–0.61 (–1.33, 0.11)
			MEF A0	–0.89 (–1.65, –0.13)
			MEF Amax	1.24 (0.42, 2.05)

HRR, heart rate recovery; HRR 1 min, number of heart beats recovered within 1 min after exercise; HRR 2 min, number of heart beats recovered within 2 min after exercise; HRR 3 min, number of heart beats recovered within 1 min after exercise; MEF A0, mono-exponential function predicted heart rate end 3-minute recovery period; MEF Amax, mono-exponential function recovered heart rate end 3-minute recovery period; SMD, standardised mean difference; T30, negative reciprocal of slope of regression line between natural logarithm of heart rate from first 30 s after exercise; T120, negative reciprocal of slope of regression line between natural logarithm of heart rate from first 120 s after exercise; T30 min, smallest time constant using negative reciprocal of slope of regression line between natural logarithm of heart rate from first 30 s after exercise;

Table S4. Methodological quality assessment of included studies judged using TESTEX scale.

	Study quality					Study reporting								
**Study**	Item 1	Item 2	Item 3	Item 4	Item 5	Item 6	Item 7	Item 8	Item 9	Item 10	Item 11	Item 12	Overall	Judgement
Audette et al. [65] *	1	0	0	1	0	0	0	1	1	0	0	1	5	Poor
Boutcher et al. [66]	1	0	0	1	1	0	1	1	1	0	0	1	7	Fair
Lopes et al. [77]	1	0	0	1	1	0	1	1	1	0	1	1	8	Fair
Melanson and Freedson [78]	1	0	0	1	1	0	0	1	1	0	0	1	6	Fair
Mourot et al. [79]	1	0	0	1	1	0	1	0	1	0	0	1	6	Fair
Oliveira-Junior et al. [80]	1	0	0	1	1	0	0	1	1	0	0	1	6	Fair
Boutcher and Stein [67]	0	0	0	1	1	0	0	1	1	0	0	1	5	Poor
de Rezende Barbosa et al. [68]	1	0	0	1	1	0	0	1	1	0	0	1	6	Fair
Hautala et al. [69]	1	0	0	1	1	1	0	1	1	0	1	1	8	Fair
Heydari et al. [70]	1	0	0	1	0	0	0	1	1	0	0	1	5	Poor
Jelinek et al. [71] *	1	0	0	0	1	0	0	1	1	0	0	1	5	Poor
Kanegusuku et al. [72] *	1	0	0	1	1	0	0	1	1	0	0	1	6	Fair
Karavirta et al. [73]	1	0	0	1	1	2	0	0	1	0	1	1	8	Fair
Karavirta et al. [74]	1	0	0	1	1	0	0	0	1	0	1	1	6	Fair
Kim et al. [75] *	1	0	0	1	1	0	0	1	1	0	0	1	6	Fair
Lee et al. [76] *	1	0	0	1	1	0	1	1	1	0	1	1	8	Fair
Rezende Barbosa et al. [81]	1	0	0	0	1	1	0	1	1	0	0	1	6	Fair
Romagnoli et al. [82] *	1	0	0	1	1	0	1	1	1	0	0	1	7	Fair
Rossi et al. [83]	1	0	0	1	1	0	0	0	1	0	1	1	6	Fair
Shen and Wen [84]	1	0	0	1	1	0	0	1	1	0	0	1	6	Fair
Shiotani et al. [85]	1	1	0	1	1	0	0	0	1	0	0	1	6	Fair
Sloan et al. [86] *	1	0	0	1	1	2	0	1	1	1	0	1	9	Good
Soltani et al. [87]	1	1	0	1	1	3	0	1	1	0	0	1	10	Good
Songsorn et al. [88]	1	1	1	1	1	3	0	1	1	0	0	1	11	Good
Tulppo et al. [89]	1	0	0	1	1	0	0	0	1	0	0	1	5	Poor
Verheyden et al. [90]	1	0	0	1	1	3	0	0	1	0	0	1	8	Fair

Item 1, eligibility criteria specified; Item 2, randomisation specified; Item 3, allocation concealment; Item 4, group similar at baseline; Item 5, blinding of assessor for flow-mediated dilation; Item 6, outcome measures assessed in 85% of patients; Item 7, intention-to-treat analysis; Item 8, between-group statistical comparisons reported; Item 9, point measures and measures of variability for all reported outcome measures; Item 10, activity monitoring in control groups; Item 11, relative exercise intensity remained constant; Item 12, exercise volume and energy expenditure;

Table S5. Heterogeneity analyses for RMSSD.

**Categorical variables**				**Test for subgroup differences**	
Moderator	**Category**	**K**	**SMD (95% CI)**	***Chi*^{2}**	***p*^{a}**
Study design	Randomised	8	0.60 (0.10, 1.10)	0.06	.812
	Non-randomised	4	0.53 (0.15, 0.90)		
Sex	Males	5	0.78 (0.33, 1.24)	1.61	.447
	Females	5	0.45 (–0.21, 1.11)		
	Mixed sample	2	0.37 (–0.10, 0.85)		
Breathing control	Yes	3	0.63 (0.20, 1.06)	0.17	.677
	No	6	0.76 (0.34, 1.17)		
Exercise type	Aerobic training	8	0.55 (0.06, 1.04)	0.23	.893
	Resistance training	2	0.71 (0.25, 1.17)		
	Combined training	2	0.62 (–0.24, 1.47)		
Aerobic training method *	HIIT	5	0.47 (0.17, 0.77)	0.12	.730
	MIT	5	0.62 (–0.19, 1.43)		

**Continuous variables**				
**Moderator**	**K**	***B* (95% CI)**	***Z***	***p***
Intervention length, weeks	12	0.04 (–0.06, 0.15)	0.85	.395
Total number of sessions	12	0.01 (–0.02, 0.05)	0.92	.356

B, *B* regression coefficient; *Chi*^{2}, chi-square statistic; CI, confidence interval; HIIT, high-intensity interval training; I^2, heterogeneity index; K, number of analysis units; LVEF, left ventricular ejection fraction; MIT, moderate intensity training; *p*, probability level associated to the absolute value of *Z* statistic for *B* regression coefficient; p^a, probability level associated to *chi-squared* statistic; SMD, standardised mean difference; *Z*, *Z* statistic for *B* regression coefficient;

Table S6. Heterogeneity analyses for HF.

**Categorical variables**				**Test for subgroup differences**	
Moderator	**Category**	**K**	**SMD (95% CI)**	***Chi*²**	***p*ᵃ**
Study design	Randomised	19	0.18 (–0.09, 0.45)	2.37	.124
	Non-randomised	4	0.53 (0.18, 0.88)		
Sex	Males	11	0.22 (–0.25, 0.69)	0.24	.889
	Females	7	0.18 (–0.12, 0.49)		
	Mixed sample	5	0.28 (0.03, 0.53)		
Breathing control	Yes	3	0.21 (–0.17, 0.58)	0.25	.615
	No	12	0.35 (–0.08, 0.79)		
Exercise type	Aerobic training	15	0.30 (0.12, 0.48)	0.84	.657
	Resistance training	3	0.26 (–0.27, 0.78)		
	Combined training	5	–0.12 (–1.02, 0.77)		
Aerobic training method *	HIIT	5	0.47 (0.17, 0.77)	1.93	.165
	MIT	15	0.15 (–0.18, 0.49)		
Sessions a week	> 3 sessions	5	0.12 (–0.16, 0.40)	0.50	.481
	≤ 3 sessions	18	0.27 (–0.03, 0.56)		

**Continuous variables**				
**Moderator**	**K**	***B* (95% CI)**	***Z***	***p***
Intervention length, weeks	23	–0.04 (–0.06, –0.02)	–3.89	<.001
Total number of sessions	23	–0.01 (–0.02, –0.01)	–3.37	.001

B, *B* regression coefficient; *Chi*², chi-square statistic; CI, confidence interval; HIIT, high-intensity interval training; I^2, heterogeneity index; K, number of analysis units; LVEF, left ventricular ejection fraction; MIT, moderate intensity training; *p*, probability level associated to the absolute value of *Z* statistic for *B* regression coefficient; *p*ᵃ, probability level associated to *chi-squared* statistic; SMD, standardised mean difference; *Z*, *Z* statistic for *B* regression coefficient;
